# A Study among the Genotype, Functional Alternations, and Phenotype of 9 *SCN1A* Mutations in Epilepsy Patients

**DOI:** 10.1038/s41598-020-67215-y

**Published:** 2020-06-24

**Authors:** Daniela Kluckova, Miriam Kolnikova, Lubica Lacinova, Bohumila Jurkovicova-Tarabova, Tomas Foltan, Viktor Demko, Ludevit Kadasi, Andrej Ficek, Andrea Soltysova

**Affiliations:** 1 Department of Molecular Biology, Faculty of Natural Sciences, Comenius University, Ilkovicova 6, Bratislava, 842 15 Slovakia; 2 Department of Pediatric Neurology, Comenius University Medical School and National Institute of Children’s Diseases, Limbova 1, Bratislava, 833 40 Slovakia; 3 Center of Biosciences, Institute of Molecular Physiology and Genetics, Slovak Academy of Sciences, Dubravská cesta 9, Bratislava, 840 05 Slovakia; 4 Department of Plant Physiology, Faculty of Natural Sciences, Comenius University, Ilkovicova 6, Bratislava, 842 15 Slovakia; 5 Institute for Clinical and Translational Research, Biomedical Research Center, Slovak Academy of Sciences, Dubravska cesta 9, 845 05 Bratislava, Slovakia

**Keywords:** Genetics, Molecular biology, Neuroscience, Neurology

## Abstract

Mutations in the voltage-gated sodium channel Na_v_1.1 (*SCN1A*) are linked to various epileptic phenotypes with different severities, however, the consequences of newly identified *SCN1A* variants on patient phenotype is uncertain so far. The functional impact of nine *SCN1A* variants, including five novel variants identified in this study, was studied using whole-cell patch-clamp recordings measurement of mutant Na_v_1.1 channels expressed in HEK293T mammalian cells. E78X, W384X, E1587K, and R1596C channels failed to produce measurable sodium currents, indicating complete loss of channel function. E788K and M909K variants resulted in partial loss of function by exhibiting reduced current density, depolarizing shifts of the activation and hyperpolarizing shifts of the inactivation curves, and slower recovery from inactivation. Hyperpolarizing shifts of the activation and inactivation curves were observed in D249E channels along with slower recovery from inactivation. Slower recovery from inactivation was observed in E78D and T1934I with reduced current density in T1934I channels. Various functional effects were observed with the lack of sodium current being mainly associated with severe phenotypes and milder symptoms with less damaging channel alteration. *In vitro* functional analysis is thus fundamental for elucidation of the molecular mechanisms of epilepsy, to guide patients’ treatment, and finally indicate misdiagnosis of *SCN1A* related epilepsies.

## Introduction

The *SCN1A* gene (MIM#182389), which is coded for the voltage-gated Na^+^ channel alpha subunit Na_v_1.1, is undeniably the most clinically relevant epilepsy gene with more than 1700 variants reported so far in various epilepsy phenotypes. The most severe phenotype associated with *SCN1A* mutations is Dravet syndrome (DS; MIM#607208), which accounts for >80% epilepsy patients carrying *SCN1A* mutations. Mutations in *SCN1A* are not limited only to DS, but have been identified also in patients with generalized epilepsy with febrile seizures plus (GEFS+; MIM#604233), which is characterized by a broad spectrum of intra-familial phenotypes that vary from asymptomatic to very severe ones, even in patients with an identical mutation^1–3^. Moreover, mutations in *SCN1A* have also been identified in some patients who present Lennox-Gastaut syndrome^4,5^, myoclonic-astatic epilepsy^6,7^, epilepsy-aphasia and epilepsy-aphasia with FS+^8^, progressive myoclonic epilepsy^9^, infantile spasms^10^ or even hemiplegic migraine^11^.

In almost 50% of epilepsy patients, *SCN1A* mutations arise *de novo*, whereas only around 9% of the patients inherit the mutation from an unaffected parent. *De novo SCN1A* mutations are observed in the severe phenotype of DS up to 60%. In comparison with others, such as GEFS+, *de novo* mutations are identified only in 3.7%^12^.

Most of the identified mutations are novel, and thus difficult to predict the type of phenotype that will develop. In DS, more than 60% of the mutations are truncating, whereas in other syndromes, more than 60% are missense with almost 90% identified in GEFS+^12^.

Truncating *SCN1A* mutations, which are mainly associated with severe phenotypes^7,12^, cause premature truncation of the sodium channel protein, indicating they will produce nonfunctional alleles and thus lead to complete haploinsufficiency of Na_v_1.1^13–15^. However, the functional effect of missense variants on channel function is more difficult to predict without functional studies. Pathogenic missense variants associated with DS, in most cases, lead to a complete loss of function, while variants associated with milder phenotypes, such as GEFS+, are known to cause milder alterations of channel function^12^.

The same missense variants may be observed in patients with various phenotypes; however, it is not fully understood why some of them are linked to specific phenotypes while others are not. Generally used genotype-phenotype correlations are difficult to accomplish due to high genotype and phenotype variability. Still, there have been attempts to bring these relationships together in order to further understand the mechanisms of epilepsy. While some variants allow for a genotype-phenotype correlation^16–18^, the spectrum of phenotypes associated with other variants is often unexplained. Functional studies aim to elucidate the impact of identified missense variants on the protein function and thus help to unravel phenotype diversity.

The aim of this study was to investigate the electrophysiological properties of nine variants (p.Asp249Glu, p.Glu788Lys, p.Thr1934Ile, p.Arg1596Cys, p.Met909Lys, p.Trp384*, p.Glu78*, p.Glu78Asp, p.Glu1587Lys), which were identified in patients suffering from various epileptic phenotypes, such as GEFS+, EE, MAS, DS, as well as to determine the possible genotype-phenotype relationships.

## Results

### Patients and clinical findings

Our aim was to functionally characterise 9 *SCN1A* variants that were identified in this study (p.Asp249Glu, p.Glu788Lys, p.Met909Lys, p.Glu1587Lys, p.Thr1934Ile), including those from our previous study^19^ (p.Trp384*, p.Glu78*) and the study by Mancardi (2006) (p.Glu78Asp)^20^, as well as p.Arg1596Cys reported by several authors^6,21–24^. We have provided detailed clinical characterisation only for patients from our cohort (Table 1).

**Table 1:** Summary of the features of the Na_v_1.1 mutants studied.

	Patient 1	Patient 2	Patient 3	Patient 4	Patient 5	Patient 6	Patient 7
Variant	c.232 G > T, p.E78*	c.747 T > G, p.D249E	c.1151 G > A, p.W384*	c.2362 G > A, p.E788K	c.2726 T > A, p.M909K	c.4759 G > A, p.E1587K	c.5801 C > T, p.T1934I
Gender	F	M	F	M	F	M	F
Age at onset	5.5 mo	7 mo	6 mo	3 mo	17 mo	7 mo	4 mo
Normal development before onset	yes	yes	yes	yes	yes	yes	yes
Seizure types	PCSG, TCS, MS	PCSG, GTCS	GTS, GTCS	PCSG, GTS	FS, 1x GTCS	MAS, GTCS	TCS (focal onset)
Family history	no	no	no	no	yes	no	no
Psychomotor retardation after 2 y of age	yes	no	yes	yes	no	yes	yes, before 2 years
Pharmaco-resistant	yes	no	yes	yes	no	yes	yes
Treatment	VPA, LEV, ETS, TPM, CBZ	VPA	VPA, PB, CLB, TPM, VNS	VPA, CBZ, PB, LTG, TPM, SLT, LEV, VNS	VPA	VPA, CLB, PRM, TPM	VPA, LEV, PRM, VGB
Hyperthermia	yes	yes	yes	NA	yes	no	yes
Phenotype	Dravet syndrome	symptoms of Dravet syndrome	Dravet syndrome	Epileptic encephalopathy with tonic generalized seizures	GEFS+	MAS	Epileptic encephalopathy
Predicted functional alteration	complete LOF	mixed effects	complete LOF	partial LOF	partial LOF	complete LOF	partial LOF
References	^19^	This study	^19^	This study	This study	This study	This study

Abbreviations: M, male; F, female; mo, month; y, year; N/A, not available; FS, febrile seizures; GEFS + , generalized epilepsy with febrile seizure plus; GTCS, generalized tonic-clonic seizures; GTS, generalized tonic seizures; LOF, loss-of-function; MAS, myoclonic-atonic seizures; MS, myoclonic seizures; PCSG, partial complex seizures with secondary generalization; TCS, tonic-clonic seizures

VPA, valproate; LEV, levetiracetam; ETS, ethosuximide; TPM, topiramate; CBZ, carbamazepine; PB, phenobarbiturate; CLB, clobazame; VNS, vagus nerve stimulation; LTG, lamotrigine; SLT, sulthiame; PRM, primidone; VGB, vigabatrine.

#### Patient 1

**Figure 1:**
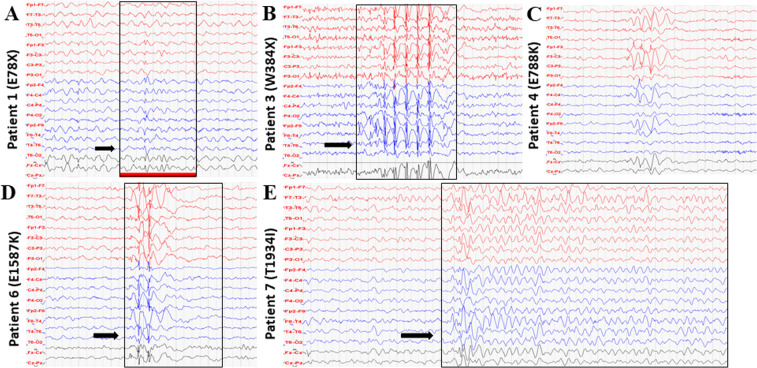
EEG recordings. (**A**) EEG record of patient 1 at four years of age showed repeated right-sided seldom diffuse background activity slowing to 2–4 Hz, and isolated spike-wave discharges with frontal and central dominance (arrow). (**B**) EEG record of patient 3 at five years of age showed bifrontal synchronous spike-wave complexes (arrow). (**C**) EEG record of patient 4 at 12 years of age showed intermittent slowing, theta waves, without epileptic graph elements. (**D**) EEG record of patient 6 at three years of age showed intermittent specific epileptic graph elements with left-side dominance (arrow). (**E**) EEG record of patient 7 at four years of age showed slow background activity 5 Hz, repeated generalized discharges of biphasic sharp waves and spike-wave complexes with delta waves (arrow).

**Figure 2:**
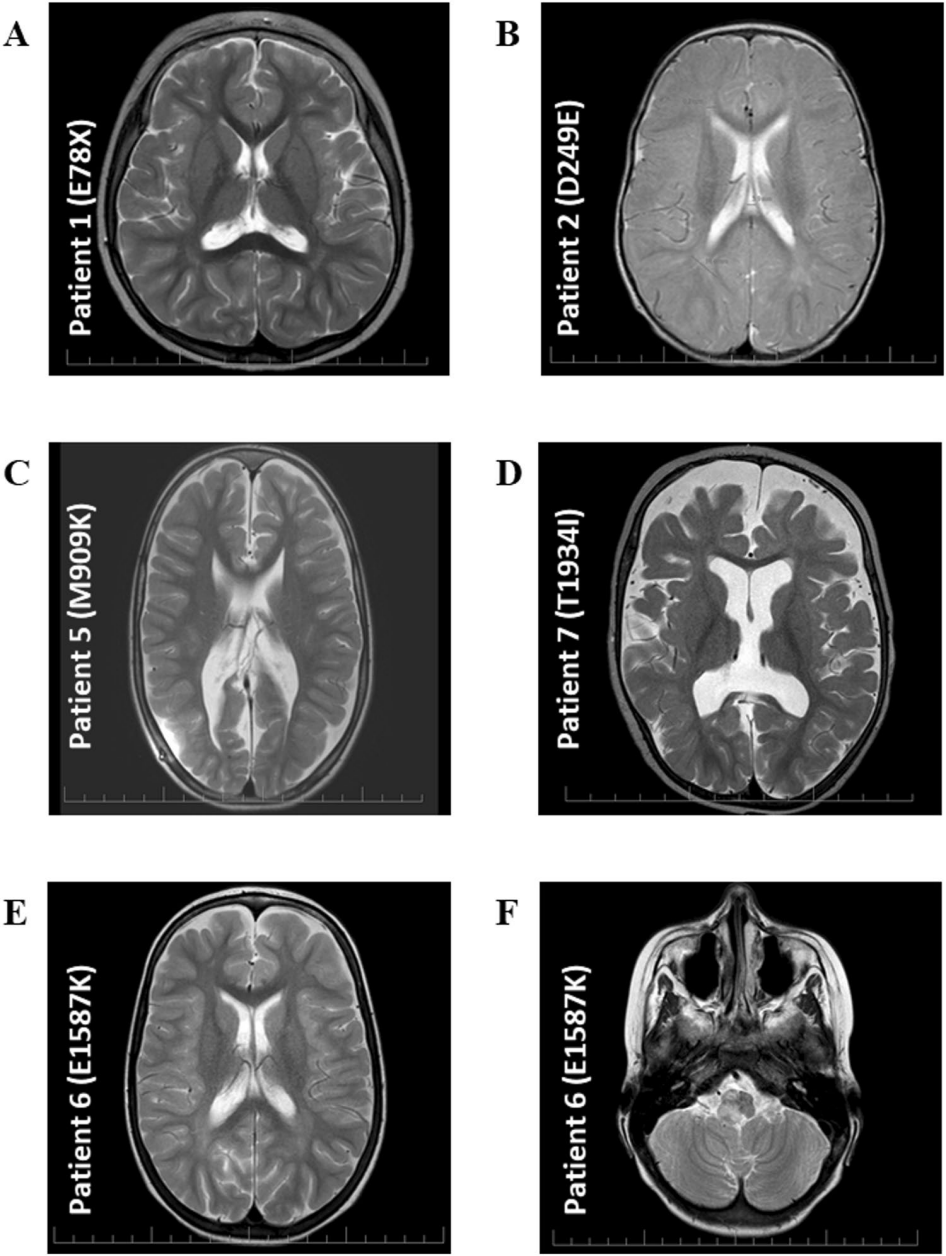
Brain MRI. (**A**) MRI of patient 1 at three years of age showed areal leukoencephalopathy in the occipital periventricular areas bilaterally, without acute changes. (**B**) MRI of patient 2 at one year of age showed areal leukoencephalopathy in the occipital periventricular areas bilaterally, without acute changes. (**C**) MRI of patient 5 at seven years of age showed dysgenesis of corpus callosum, hydrocephalus on V-P drainage. (**D**) MRI of patient 7 at one year of age showed progressive diffuse brain atrophy. (**E,F**) MRI of patient 6 at six years of age showed frontal and occipital areas of leukoencephalopathy, tumor in the brainstem.

A 12 year-old girl who was diagnosed with symptoms of Dravet syndrome at the age of five and a half months, with normal psychomotor development before seizure onset. She exhibited complex partial seizures with secondary generalisation and later on, tonic-clonic and myoclonic seizures resistant to pharmacological treatment. Developmental delay and ataxia developed during the disease. EEG showed repeated right-sided seldom diffuse background activity slowing to 2–4 Hz, and isolated spike-wave discharges with frontal and central dominance (Fig. 1A). Brain MRI showed no significant signal changes (Fig. 2A). Genetic analysis revealed mutation p.E78*.

#### Patient 2

A five year-old boy who was diagnosed with symptoms of Dravet syndrome at the age of seven months with normal psychomotor development before seizure onset. He exhibited partial complex seizures and later on, generalised tonic-clonic seizures associated with fever. EEG was normal up to 20 months of age, then spike and wave complexes began to appear. MRI showed areal leukoencephalopathy in occipital periventricular areas bilaterally without acute changes (Fig. 2B). The patient has an atactic gait. Genetic analysis revealed variant p. D249E.

#### Patient 3

A 12 year-old girl who exhibited generalised tonic and tonic-clonic seizures associated with fever at the age of six months with developmental delay later during the disease. EEG showed bifrontal synchronous spike-wave complexes (Fig. 1B) and MRI showed left-side temporal lobe hypotrophy with secondary CSF pathway dilatation (not available). Her seizures are pharmacoresistant and non-pharmacological treatments include VNS. Genetic analysis revealed mutation p. W384*.

#### Patient 4

An 18 year-old boy who exhibited partial complex seizures with generalization and primarily generalized tonic seizures at 3 months of age. EEG showed intermittent slowing, theta waves, absence of epileptic graph elements (Fig. 1C); epileptic EEG changes started at the age of seven. Brain MRI showed no significant signal changes (not available). Like the previous patient, he is pharmacoresistant and was implemented with VNS. He was diagnosed with epileptic encephalopathy with generalized tonic seizures. Genetic analysis revealed variant p. E788K.

#### Patient 5

A nine year-old girl with a positive family history of epilepsy, who exhibited repeated febrile seizures at the age of 17 months and later on, a single generalized tonic-clonic seizure at five years. EEG was normal and MRI showed dysgenesis of corpus callosum, hydrocephalus on V-P drainage (Fig. 2C), comorbidities including meningomyelocoele. She was diagnosed with GEFS+, and genetic analysis revealed variant p. M909K inherited from the mother, who also exhibited seizures.

#### Patient 6

An eleven year-old boy who exhibited myoclonic-atonic seizures and generalized tonic-clonic seizures in his sleep. No prior psychomotor development delay was observed, however, developmental delay occurred during the disease. EEG showed intermittent specific epileptic graph elements with left-side dominance (Fig. 1D). Brain MRI showed frontal and occipital areas of leukoencephalopathy and tumour in the brainstem (Fig. 2E,F). He is pharmacoresistant and diagnosed with myoclonic-atonic seizures. Genetic analysis revealed *de novo* variant p. E1587K.

#### Patient 7

A nine year-old girl who exhibited focal seizure onset, tonic-clonic seizures at 4 months of age. Psychomotor development delay was observed after seizure onset. EEG showed slow background activity 5 Hz, repeated generalized discharges of biphasic sharp waves and spike-wave complexes with delta waves (Fig. 1E). MRI displayed progressive diffuse brain atrophy (Fig. 2D). She is pharmacoresistant and diagnosed with epileptic encephalopathy. Genetic analysis revealed variant p. T1934I inherited from the unaffected father.

### Prediction of mutation impact and evolution conservation

**Figure 3:**
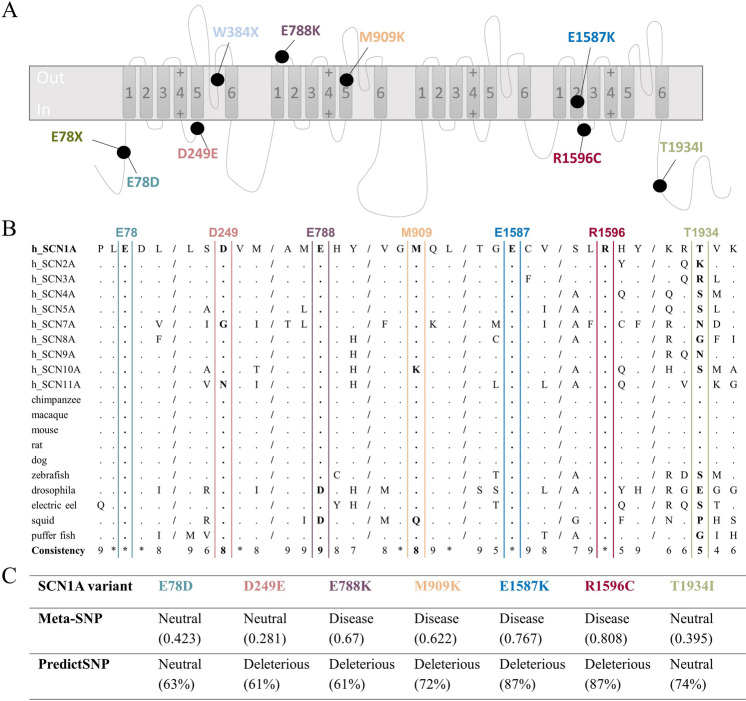
Overview of the studied mutants – localisation, evolution conservation, variant prediction. (**A**) Topology diagram of *SCN1A* variants. Schematic representation of the sodium channel molecular complex illustrating the location and amino acid substitution of the studied *SCN1A* variants. (**B**) The amino acid sequence alignment of the Na_v_ α-subunit family, showing the evolutionary conservation of the residues (boxed). E78, D249, E788, M909, E1587, R1596, and T1934 are shown in bold. Residues that are identical to the *SCN1A* sequence are displayed as dots. The conservation scoring was performed by PRALINE. The scoring scheme works from 0 for the least conserved alignment position, up to 10 for the most conserved alignment position. Conservation in all studied sequences is marked as *. (**C**) The results of *SCN1A* variant prediction by Meta-SNP and PredictSNP. “Neutral” referred to a neutral variant, “Disease” and “Deleterious” to disease-causing variants. The variant was predicted to be disease-causing when Meta-SNP predictions were >0.5. The percentages are the expected prediction accuracies by PredictSNP.

The studied variants, located in various domains of hNa_v_1.1 (Fig. 3A, Supplementary Table 1), were tested with Meta-SNP and PredictSNP tools, which combine several programmes to predict the impact of amino acid substitution more accurately. Variants E788K, R1596C, M909K, and E1587K were classified as pathogenic, and E78D and T1934I as neutral by both prediction tools. Variant D249E was classified as neutral by Meta-SNP, however, it was characterized as pathogenic by PredictSNP. The results are given in Fig. 3C, which summarizes the evaluation from individual tools covered by both approaches.

PRALINE programme was used to evaluate the evolutionary conservation of aligned amino acid sequences of the Na_v_ α-subunit family. E78, E1587, and R1596 are highly conserved in all ion channels. D249, E788, and M909 are highly conserved throughout the presented Na_v_ members with scores 8, 9 and 8 respectively, while T1934 had a score of 5 (Fig. 3B).

### Biophysical characterization of *SCN1A* variants

**Figure 4:**
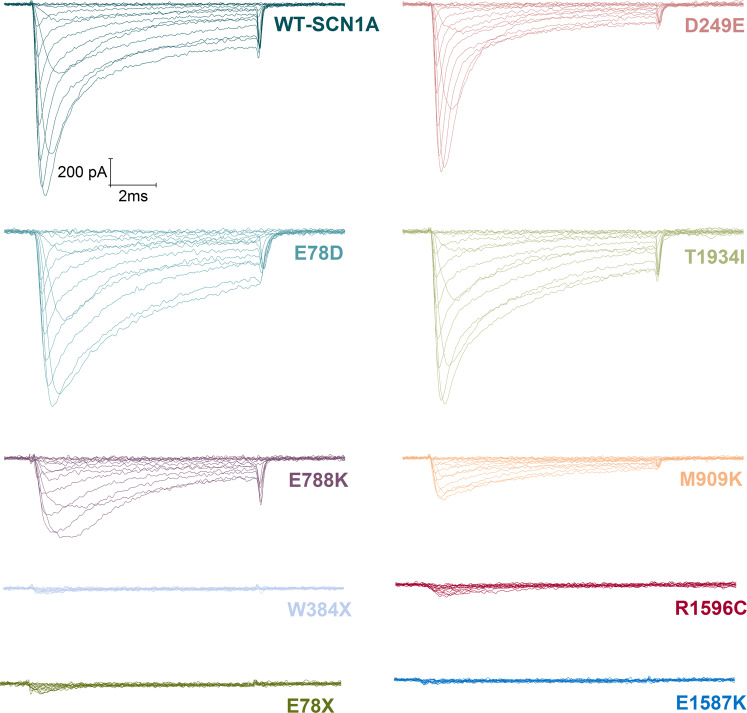
Representative current traces measured from WT and mutant whole-cell *SCN1A* sodium currents. Whole-cell sodium currents were recorded from HEK293T cells transiently expressing the indicated alleles during voltage steps to potentials varying between −70 and +60 mV with a 10 mV increment from a holding potential of −100 mV. Vertical and horizontal scale bars represent 200 pA and 2 ms, respectively.

In order to characterize the biophysical properties of *SCN1A* variants, we transiently transfected wild-type (WT) and mutant Na_v_1.1 channels into HEK293T cells. Sodium currents were measured using whole-cell patch-clamp recording of heterologously expressed recombinant hNa_v_1.1 sodium channels. Four out of nine mutants, E78*, W384*, E1587K, and R1596C failed to produce sodium currents similarly to endogenous currents present in untransfected HEK293T cells (data not shown), which suggests that they are complete loss-of-function mutants in contrast to recordings from the cells transfected with hNa_v_1.1-WT (Fig. 4). The non-functional alleles, E78*, W384*, E1587K, and R1596C affect residues located within the N-terminal region, DI pore loop, DIVS2 segment, and DIVS2-S3 segment respectively (Fig. 3A, Supplementary Table 1). By verifying the complete coding sequence of each mutant construct, we ruled out any cloning artefacts that may cause non-functional sodium channels.

**Figure 5:**
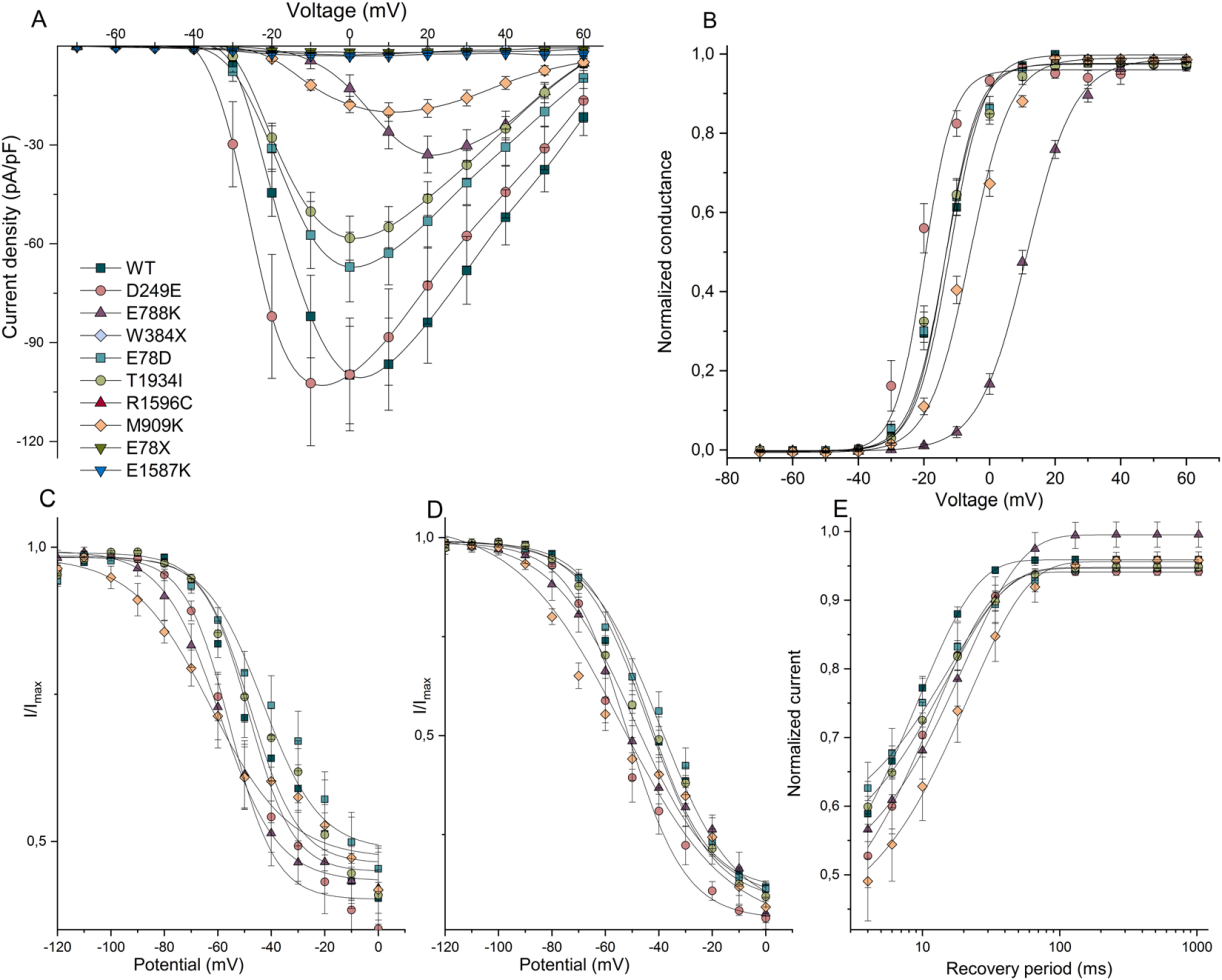
Functional properties of wild-type and mutant channels. (**A**) I-V relationships of whole-cell currents from transiently transfected cells. Current amplitudes were normalised to cell capacitance and plotted against test potentials. Solid lines are connectors of experimental data. (**B**) voltage dependence of current activation. The voltage dependence of channel activation was estimated from G-V relationships. Individual G-V relationships were normalized with respect to corresponding maximal conductance of each cell. (**C**) voltage dependence of steady-state fast current inactivation. Currents were normalised with respect to the maximal current amplitude of each cell. (**D**) voltage dependence of current inactivation evoked by prolonged 1 s conditioning prepulse normalised in respect to the maximal current amplitude of each cell. (**E**) recovery from steady-state fast inactivation. Solid lines are mean fits of single exponential relationships of the data. Solid lines in panels B,C,D represent fits of experimental data by Boltzmann relationships. Individual symbols are explained in panel A.

Five mutants (D249E, E788K, E78D, T1934I, and M909K), which encoded partially functional channels, were further characterized. In cells transfected with E78D, D249E, and T1934I, sodium currents were similar in amplitude to hNa_v_1.1-WT without normalization of cell capacitance (Fig. 4), however, significantly reduced after normalization only in T1934I cells (p < 0.05) (Fig. 5A). Figure 4 illustrates representative whole-cell sodium currents evoked by a series of depolarizing voltage pulses in cells expressing either WT-SCN1A or each of the nine mutant channels.

Current-voltage relationships (Fig. 5A) illustrate that mutant channels exhibited significantly lower (E788K, CD = 33 ± 6; p < 0.005; W384* CD = 3 ± 0.4; p < 0.0001; T1934I, CD = 58 ± 7; p < 0.05; R1596C, CD = 3 ± 1; p < 0.0001; M909K, CD = 20 ± 3; p < 0.0001; E78*, CD = 2 ± 0.5; p < 0.0001; and E1587K, CD = 3 ± 0.6; p < 0.0001) peak sodium current densities when compared to WT-SCN1A (CD = 100 ± 15), while peak current densities of D249E (CD = 102 ± 19), and E78D (CD = 67 ± 11) were not significantly changed (Table 2).

**Table 2:** Functional parameters of sodium currents recorded and predicted influence of biophysical properties on channel activity based on observed changes.

		WT-SCN1A	D249E	E788K	E78D	T1934I	M909K
CD (pA/pF)		100 ± 15	102 ± 19	**33** ± 6**	67 ± 11	**58** ± 7*	**20** ± 3***
Voltage dependence of activation	V_1/2_ (mV)	−13.7 ± 0.7	**−21.3** ± 1.5***	**11.1** ± 0.6***	−14.1 ± 1.4	−14.3 ± 1.3	**−6.4** ± 1.1***
	V_1/2_ ∆ (mV)		**−7.6** ± 0.8*** ↑	**24.8** ± 0.1*** ↓	−0.4 ± 0.7	−0.6 ± 0.6	**7.3** ± 0.4*** ↓
	k (mV)	6.6 ± 0.2	**5.1** ± 0.6*	7.4 ± 0.8	6.3 ± 0.6	6.6 ± 0.4	7.0 ± 0.4
	k ∆ (mV)		1.3* ↑	0.9	1.0	1.0	0.9
	n	21	9	11	11	12	14
Voltage dependence of steady-state fast inactivation	V_1/2_ (mV)	−43.7 ± 3.0	**−56.6** ± 2.5*	**−58.4** ± 2.2*	−39.0 ± 3.9	−43.2 ± 2.4	**−62.4** ± 4.3**
	V_1/2_ ∆ (mV)		**−12.9** ± 0.5* ↓	**−14.7** ± 0.8* ↓	4.7 ± 0.9	0.5 ± 0.6	**−18.7** ± 1.3** ↓
	k (mV)	12.3 ± 1.0	9.4 ± 1.4	11.1 ± 0.9	13.4 ± 1.0	13.8 ± 1.6	15.5 ± 2.9
	k ∆ (mV)		1.3	1.1	0.9	0.9	0.8
	Noninactivating part of the current	40 ± 3%	35 ± 5%	42 ± 6%	45 ± 4%	41 ± 4%	42 ± 7%
	n	20	9	5	13	12	6
Voltage dependence of current inactivation evoked by prolonged 1 s conditioning prepulse	V_1/2_ (mV)	−44.2 ± 2.5	−52.3 ± 3.9	−50.9 ± 4.2	−39.2 ± 2.9	−43.2 ± 4.0	−53.9 ± 4.1
	V_1/2_ ∆ (mV)		−8.1 ± 1.4	−6.7 ± 1.7	5.0 ± 0.4	1.0 ± 1.5	−9.7 ± 1.6
	k (mV)	12.3 ± 0.6	11.4 ± 1.5	**16.8** ± 1.8*	13.8 ± 0.9	14.2 ± 1.3	**19.8** ± 3.8**
	k ∆ (mV)		1.1	0.7* ↓	0.9	0.9	0.6** ↓
	Noninactivating part of the current	12 ± 0.01%	**4** ± 0.01%**	**5** ± 0.04%*	11 ± 0.02%	9 ± 0.03%	**7** ± 0.01%*
	n	24	8	5	13	12	6
#		***	***	**	***	***	**
Recovery from fast inactivation	τ (ms)¶	9.0 ± 0.4 [59 ± 4%]	**12.0** ± 1.5* ↓ [61 ± 7%]	**19.8** ± 2.1*** ↓ [53 ± 4%]	**17.0** ± 3.7*↓ **[46** ± 5%]*	**16.8** ± 2.6** ↓ [48 ± 5%]	**24.3** ± 5.5*** ↓ [60 ± 7%]
	n	23	9	5	13	13	8

Abbreviations: CD, current density; V1/2, voltage of half-maximal activation or inactivation; V1/2 ∆, values represent shift of voltage for half-maximal activation or inactivation relative to the value measured from the wild type channel; k, slope factor of the activation or inactivation curve; k ∆, values indicate multiple of slope factor of the activation or inactivation curve measured from wild type channel. Original values are listed in the column representing the wild type channel. #, represents significant difference between parameter of noninactivating part of the current characterizing steady-state fast inactivation and the same parameter characterizing inactivation evoked by prolonged 1 s conditioning prepulse of the same channel construct; τ represents time constant of monoexponential fit of data from recovery from steady-state fast inactivation; ¶ values in square brackets represent a relative amplitude of inactivated part of the current; ↑, predicted gain of channel activity; ↓, predicted loss of channel activity

Values statistically significantly different from WT-SCN1A are printed in bold and indicated as follows: *p < 0.05; ** p < 0.005; *** p < 0.0001.

The peak sodium current density was reached at a membrane voltage that was shifted by individual mutations. Current-voltage (I-V) relations for mutants D249E, E788K, and M909K reached the maximum at −10 mV, +20 mV, and +10 mV, respectively, while I-Vs for other mutants and WT-SCN1A peaked at 0 mV (Fig. 5A).

### Disturbances in voltage dependencies of current activation and inactivation

Shifts of I-V relationships along the voltage axis (Fig. 5A) were evident for some mutants. Therefore, we analyzed voltage dependencies of activation and inactivation in more detail.

The experimental data presented in Fig. 5B were fitted by the Boltzmann equation (see Methods). The resulting values document that half-maximal activation voltages of three mutants were significantly shifted in either the hyperpolarized (D249E, V_1/2_ = −21.3 ± 1.5; p < 0.0001) or depolarized direction (E788K, V_1/2_ = 11.1 ± 0.6; p ˂ 0.0001, and M909K, V_1/2_ = −6.4 ± 1.1; p ˂ 0.0001; Fig. 5B, Table 2) when compared to the half-maximal activation (V_1/2_) of WT-SCN1A (V_1/2_ = −13.7 ± 0.7). Additionally, the slope factor of activation of D249E mutant significantly decreased, suggesting increased voltage sensitivity of the channel (Table 2). These findings indicate that E788K and M909K sodium channels require greater depolarization to activate, while activation of the D249E requires smaller depolarization, thus possibly contributing to altered neuronal excitability.

The voltage dependence of steady-state fast inactivation was significantly shifted in the hyperpolarizing direction. Voltages for half-maximal inactivation (V_1/2_) were D249E, V_1/2_ = −56.6 ± 2.5; p < 0.05; E788K, V_1/2_ = −58.4 ± 2.2; p < 0.05; and M909K, V_1/2_ = −62.4 ± 4.3; p < 0.005 (Fig. 5C, Table 2) when compared to WT-SCN1A (V_1/2_ = −43.7 ± 3.0).

Differences in voltage dependencies of inactivation evoked by prolonged 1 s inactivating prepulses were less remarkable (Fig. 5D). No significant differences were found in the V_1/2_ values between mutant and wild-type channels. However, slope factor of voltage dependence of inactivation evoked by prolonged 1 s inactivating prepulses was significantly enhanced in E788K, k = 16.8 ± 1.8; p < 0.05; and M909K, k = 19.8 ± 3.8; p < 0.005 when compared to the slope value of WT-SCN1A (k = 12.3 ± 0.6), thereby suggesting reduced voltage sensitivity of channels (Table 2).

Data from experiments that assess recovery from steady-state fast inactivation were fitted with a single exponential association function, since recovery closely followed a curve with one time constant. All mutations significantly slowed kinetics of recovery from inactivation (D249E, τ = 12.0 ± 1.5 ms; p < 0.05; E788K, τ = 19.8 ± 2.1 ms; p < 0.0001; E78D, τ = 17.0 ± 3.7 ms; p < 0.05; T1934I, τ = 16.8 ± 2.6 ms; p < 0.005; M909K, τ = 24.3 ± 5.5 ms; p < 0.0001) when compared to WT-SCN1A (τ = 9.0 ± 0.4 ms). (Fig. 5E, Table 2).

## Discussion

The importance of *SCN1A* gene mutation screening in severe epilepsy syndromes, such as Dravet syndrome is well established, however, the role of *SCN1A* variations in other phenotypes is not fully understood^4–10^. There are few studies focused on the phenotype-genotype correlation based on the clinical significance of specific *SCN1A* mutations, mutation class, and amino acid localization and its properties^7,25^. Nevertheless, a clearly defined phenotype-genotype correlation in *SCN1A*-related epilepsies other than Dravet or GEFS+ are non-existent, most likely due to a lack of data.

The present study was designed to determine the basic biophysical properties of nine variants identified in the human neuronal voltage-gated sodium channel gene *SCN1A* in patients with various inherited epilepsy syndromes, and later compare them with the clinical phenotypes.

The two studied nonsense mutations, E78X and W384X, as well as two missense mutations, E1587K and R1596C, encoded channels with complete loss-of-function. The remaining five *SCN1A* variants were functional channels that exhibited a wide range of alterations in functional properties.

Altered voltage dependence of activation with either hyperpolarizing (D249E) or depolarizing shift (E788K and M909K) was observed in cells which expressed three of the studied alleles. These three mutations also caused hyperpolarizing shifts in the voltage dependence of fast steady-state inactivation.

Allele D249E caused opposing changes in functional properties. The hyperpolarizing shift of activation and reduced slope factor of activation are consistent with gain-of-function, while a hyperpolarizing shift of inactivation, along with slower time constant of recovery from inactivation, represent loss-of-function features. The shift in the voltage dependence of inactivation was greater than the corresponding shift in activation, thus indicating that the window current, which is defined by the area of overlap between activation and inactivation curves, was reduced in the mutant D249E compared to WT. However, such opposing changes make it difficult to predict the overall impact of the D249E mutation on neuronal excitability.

Both of the alleles E788K and M909K resulted in a depolarizing shift of activation and hyperpolarizing shifts of inactivation. The voltage dependence of activation in E788K was greater than the shift of inactivation, while in M909K, voltage dependence of inactivation was greater. Both E788K and M909K displayed significantly reduced current density, reduced voltage sensitivity, and slower recovery from inactivation. All these alterations suggest that E788K and M909K are partial loss of function mutations, with M909K likely having a greater impact on hNav1.1 function. Since the Na_v_1.1 is expressed alone in this study, and it has been shown that some mutants can be rescued by interacting proteins that likely stabilize the folding/trafficking defects^26–29^. We assume that mutant M909K associated with GEFS+ phenotype may be partially rescued in some conditions, whereas E788K in the patient with epileptic encephalopathy is probably not rescued.

On the basis of functional properties, alleles E78D and T1934I are said to be the least severe ones. Both alleles slowed kinetics of recovery from inactivation, however, significantly reduced current density was observed only in T1934I.

Dravet syndrome was diagnosed in patients with mutations E78X, E78D^20^, D249E, and W384X. Mutation W384X was described previously^6^ in a female patient with SMEB-O, with the onset at 6 months of age, the same as the patient in this study. Just as we presumed, both alleles with nonsense mutations encoded nonfunctional channels, which is in correlation with the patients’ severe phenotype. As previously reported^30^, the pathological impact of nonsense mutations is caused by a lack of important channel domains, which leads to haploinsufficiency as the cause of DS^31^. *De novo* variant E78D, which was predicted as neutral by *in silico* predictors and located in the same position p.Glu78 as E78X, was functionally characterized with only slower recovery from inactivation. The clinical severity of DS predominantly expects variants to show the biophysical characteristics of loss-of-function defects, however, few studies have so far identified mutations with measurable sodium currents. The previously studied mutations Y426N and T1909I, which were identified in DS patients, revealed significantly lower current densities^32^ in addition to other biophysical abnormalities. Similarly, the alleles R1648C and F1661S were observed to have altered gating properties and significantly lower current density in cells expressing F1661S. The reduced current density was the only abnormality detected in G1749E^33^. Based on these studies, *SCN1A* channels that carry mutations associated with DS can be either non-functional or functional, thus showing a broad range of abnormalities that indicate the complex molecular and cellular basis for this disorder. Moreover, genetic, metabolic and environmental factors, as well as interacting proteins are supposed to modify the clinical expression of the molecular defect in each patient. A comparison of the biophysical findings in DS patient with D249E mutation corresponds to observations of other studies where the alterations towards hyperpolarizing potentials in activation and inactivation for mutation W1204R was identified in the family with GEFS+^34,35^, and GEFS+ with DS^26^. Similar results were observed for GEFS+ mutant R859H with mixed biophysical gating defects^36^, and mutation T226M with the voltage dependence of activation and inactivation shifted towards hyperpolarizing potentials^37^, however, patients harbouring this mutation suffered from early infantile *SCN1A* encephalopathy, a condition different from DS. These patients had pharmacoresistant early-onset hemiclonic seizures that occurred at a mean age of 9 weeks, and tonic-clonic seizures by 18 months; additional symptoms included developmental delay, intellectual disability, and movement disorder^18^.

Variant T1934I, predicted by *in silico* predictors as neutral and with slower recovery from inactivation, thereby significantly reducing current density detected in the functional analysis, was found in the patient manifested with focal onset, tonic-clonic seizures at 4 months with serious EEG and MRI findings (Figs. 1E, 2D) and diagnosed with epileptic encephalopathy. Parental genetic analysis revealed that the unaffected father was carrying the same variant. Taken together, and based on the PRALINE results which predicted conservation of Thr at position 1934 with score 5 (Fig. 3B,C), the finding does not correspond to the patient´s severe phenotype. These results provide further support for the hypothesis that T1934I variant may be a modifying allele that correlates only with some of the symptoms, and/or this patient has a pathological mutation in an additional gene. Indeed, subsequent WES analysis of this patient revealed pathological *de novo* mutation in the *SMC1A* gene (data not shown), hence the functional study of this variant indicated the misdiagnosed problem of *SCN1A*-related epilepsies.

Variant E788K, which was identified in the patient with epileptic encephalopathy with tonic generalized seizures, and also M909K in the patient with GEFS+, revealed similar biophysical alterations. The positive shift of activation and the negative shift of inactivation was previously described for allele V983A in a patient with intractable childhood epilepsy with generalized tonic-clonic seizures (ICEGTC), along with reduced peak current density^38^. In the GEFS+ family with mutation I1656M, the same shifts in activation and inactivation were observed, however, current density was not reduced when compared to the WT channel^34^. These observations indicate the complexity of the phenotype formation, where similar biophysical alteration may lead to different clinical manifestations. As discussed above, the interacting proteins may potentially rescue the mutant M909K *in vivo* and modulate the phenotype to milder GEFS+, however, in the case of E788K mutant, the lack of rescue *in vivo* could result in severe phenotype.

*De novo* variant E1587K was identified in the boy with MAE, who exhibited myoclonic-atonic seizures and generalised tonic-clonic seizures in sleep. This allele encoded for nonfunctional sodium channels. Few cases had reported mutations in *SCN1A* in patients with MAE^6,7,39,40^, however, none of the mutations had been functionally tested.

Allele R1596C is a variant that expresses into many phenotypes and was first identified in a boy with suspected diagnose of GEFS+^22^. The same amino acid change was reported in a patient with cryptogenic focal epilepsy (CFE) with various seizure types, such as generalized tonic-clonic seizures, myoclonic jerks, focal, and tonic seizures^6^. Later, it was identified in a patient with typical DS^21^ and also in atypical multifocal DS characterized by the absence of generalized seizures and later developmental slowing^24^. Hoffman-Zacharska^23^ studied R1596C in a family with five probands carrying this variant, in which one of them was asymptomatic, one suffered from febrile seizures plus (FS+), two probands were diagnosed with the phenotype of epilepsy with GTCS and one with atypical DS. Since the variant is linked to such a variety of phenotypes, we aimed to study its net functional effect. Patch-clamp recordings in HEK293T cells revealed completely nonfunctional sodium channels in a heterologous expression system. We assume that the variation in clinical phenotypes is not related only to the mutation itself, but rather to the interaction with other genetic or environmental factors^41–45^. Since the mutation R1596C may develop into phenotypes with various severity there is a possible explanation, that this variant could be rescued by interacting proteins *in vivo* leading to the milder phenotypes described in the literature and, conversely, lack of rescue *in vivo* could result in complete LOF in patients with DS and other severe epileptic phenotypes. That means the outcome of this mutation could be dependent on the genomic background of each patient.

The study aimed to contribute to the understanding of biophysical changes of the functionally studied variants in various positions of the Na_v_1.1 protein identified in patients with different phenotypes in order to better understand the pathogenesis of epilepsy and variation of clinical expression of *SCN1A* mutations. Pathogenicity of *SCN1A* mutations varies, since truncating and missense mutations which cause LOF occurred more frequently in patients with severe phenotypes, while missense variants with detectable sodium currents, regardless of their biophysical properties, indicate milder phenotypes. Mutations in the pore region (S5-S6) were mostly associated with LOF, nonetheless, those occurring in other regions were either LOF or partial LOF and with mixed effects of gain and loss of function^12^.

The results of this study do not explain the complexity between clinical phenotype and biophysical properties, however, functional studies in heterologous expression system are still a very effective model for generation of reasonable data in order to study the initial effects of *SCN1A* mutations. Still, there is a need to identify and standardize experimental conditions. In some mutants, we can correlate the net gating properties measured in *in vitro* transfected cells with the severity of phenotypes, however, in cases where the same alterations were identified in individuals with milder as well as severe phenotypes, we have to take into account the possible modifying mechanisms. For example, expression in a neuronal cell background can partially rescue mutant channels, as it was seen for familial hemiplegic migraine (FHM) mutations. Expression in neurons could mimic real conditions regarding the cell background. A study of FHM Na_v_1.1-L1649Q revealed that it is a loss of function mutant caused by folding/trafficking defects and can be rescued by interacting proteins or by expression in a neuronal cell background^46^. Another folding/trafficking defective FHM mutation, Na_v_1.1-L1670W, showed that functional effect can be switched from a complete loss of function to the gain of function by expression in neurons^47^. Epileptogenic mutations cause a variable degree of loss of function, whereas FHM mutations can, in some cases of functional studies, appear as loss of function because of rescuable folding/trafficking defects; other studies, however, confirmed that FHM mutations cause a gain of function of Na_v_1.1^48,49^. Several studies have also suggested that environmental factors and genetic modifiers may influence the clinical phenotype of *SCN1A*-related epilepsy^41–45,50,51^. Further studies of the effects of channel mutations in the context of all the other variants in the patient’s genome using patient-derived induced pluripotent stem cells (iPSCs) may be useful^52–55^. However, these findings may be somewhat limited by conflicting results, showing contradictory changes in sodium currents and excitability in neurons derived from iPSCs of Dravet syndrome patients. It is crucial to obtain the full maturation of differentiated iPSC-derived neurons, which can be a challenge to obtain *in vitro*. Differences in functional changes due to neurons at a different stage of maturation may be the explanation, as well as the large functional heterogeneity between neurons in the same neuronal culture. The iPSC technology is still expensive and time consuming, and technical improvements are necessary.

## Material and methods

### Molecular SCN1A analysis

Genomic DNA was isolated from peripheral blood samples using the GENTRA PureGene Blood Kit (Qiagen, Germany) according to the manufacturer’s recommendations. All 26 coding exons of the *SCN1A* gene (ENSG00000144285) were amplified using HOT FIREPol DNA Polymerase (Solis BioDyne, Estonia) with specific primers (primer sequence available on request) and subsequently sequenced using ABI Prism 3130xl Genetic Analyzer (Life Technologies, USA). Raw data were analyzed using the Sequencing Analysis Software v5.3 (Life Technologies, USA) with a subsequent alignment to the reference sequence (ENST00000303395) and variant identification via ChromasPro Software v1.6 (Technelysium Pty Ltd, Australia). All methods were carried out in accordance with relevant guidelines and regulations for involving human participants in the study and were approved by Institutional Ethics Committee of National Institute of Children Diseases, Slovakia. Informed consent for clinical examinations and DNA diagnostics was obtained from all participants and/or their legal guardians.

### Plasmids and mutagenesis

The cDNA for human Na_v_1.1 Na^+^ channel α subunit was provided by GlaxoSmithKline plc and encodes the shorter splice variant isoform of 1998 amino acids, which could be the predominant Na_v_1.1 variant expressed in the brain^56,57^. cDNA was subsequently cloned in order to generate pCDM8-hNav1.1 by M. Mantegazza^58^. The plasmid was propagated in TOP10/P3 cells (Thermo Fisher Scientific, USA) at 28 °C in order to minimize spontaneous rearrangement. We generated the construct pCDM8- hNa_v_1.1-EGFP in order to track plasmid in cells by the following strategy. In the first step, we amplified the plasmid pCDM8- hNa_v_1.1 by PCR with the following primers: 5´-CAACCGGTTGACAGATAGGCGGCCGCAGTGGTGGAATGCCTTTAATGA -3´ (forward, which introduced the restriction sites for AgeI and NotI) and 5´-GCTCCCATTCATCAGTTCCATAG-3´ (reverse). Fragment *EGFP* (735 bp), which was digested with AgeI and NotI from plasmid pEGFP-N1, was subcloned to the modified construct in order to obtain the plasmid pCDM8- hNa_v_1.1-EGFP, which expresses both the protein of interest and *EGFP* as a reporter. We want to emphasise that *EGFP* is not tagged to hNa_v_1.1 protein. Point mutations for each mutant were introduced using Phusion High-Fidelity DNA Polymerase (New England Biolabs, Ipswich, MA, USA) using the primers showed in Table S1. The entire coding sequence of the hNa_v_1.1 cDNA was sequenced after each propagation in order to confirm the presence of the introduced mutation and the absence of spurious mutations.

### Cell culture and transfections

HEK-293T (ATCC CRL-11268) cells were cultured in Dulbecco’s Modified Eagle Medium supplemented with 10% fetal bovine serum, 10 units/mL penicillin and 10 μg/mL streptomycin (Merck, USA) and grown at 37 °C with 5% CO_2_. For electrophysiological recordings, cells were transiently transfected with plasmids carrying Na_v_1.1-WT or mutants (1 μg) in 12-well culture plates using Lipofectamine 3000 (Thermo Fisher Scientific, USA). Cells were harvested after 16–20 h and plated in 24-well culture plates onto 13-mm round coverslips coated with 0,01% poly-L-lysine. Recordings were made 2–3 days after transfection.

### Electrophysiology and data analysis

Transfected cells were selected visually by their fluorescence. Sodium currents of WT and mutated channels were recorded at room temperature (22–25 °C) using the whole-cell configuration of the patch-clamp technique with the HEKA-10 patch-clamp amplifier (HEKA Electronic, Lambrecht, Germany).

The extracellular recording solution contained (in mM): 105 NaCl, 2 CaCl_2_, 0.5 MgCl_2_, 10 HEPES, 25 TEA-Cl, and 10 glucose, pH 7.4 titrated with NaOH. The pipette solution contained (in mM): 135 CsCl, 3 EGTA, 2 MgCl_2_, 20 TEA-Cl, 5 Na_2_-ATP, and 10 HEPES, pH 7.4 titrated with CsOH. Pipette resistance was between 1.5 and 2.5 MΩ and series resistance was between 2.5 to 5 MΩ, and was compensated up to 70%. The capacitance of cells ranged between 10 and 25 pF. The remaining linear capacity and leakage currents were subtracted by P/4 procedure. Data were recorded with HEKA PatchMaster v90.2 and analysed off-line using HEKA FitMaster v2x73.5 and Origin Pro software (OriginLab, Northampton, MA, USA). All experiments were measured from a holding potential (HP) of −100 mV.

Current–voltage (I–V) relationships of sodium inward currents were measured by a series of 10 ms long depolarizing pulses applied from HP to membrane potentials ranging from −70 to +60 mV with 10 mV increment. The voltage dependence of steady-state current inactivation (SSI) was recorded by two protocols with different length of conditioning prepulse. The fast SSI was measured by a series of 100 ms long conditioning prepulses to potentials ranging from −120 to 0 mV with 10 mV increment, followed by 5 ms long test pulses to 0 mV. In second experimental protocol 1000 ms long conditioning prepulses were used.

The conductance-voltage (G-V) relationships (activation curves) were determined from peak current (I) versus voltage relationships, using the formula G = I/(V−V_Rev_), where V was the test potential and V_Rev_ was an apparent reversal potential evaluated by linear extrapolation of experimental data according to Boltzmann-Ohm equation.

Normalized activation and inactivation curves were fit to Boltzmann relationships in the form: y = 1/{1 + exp [(V−V_1/2_)/k]}, where y is normalized G_Na_ or I_Na_ measured at the membrane potential V, V_1/2_ is the voltage of half-maximal activation or inactivation, and k is a slope factor.

Recovery from inactivation was examined by applying a 5 ms conditioning pulse to 0 mV from a holding potential of −100 mV, followed by a 100 ms long prepulse to 0 mV and a recovery interval of variable duration (Δ2 ms) and a test pulse to 0 mV. The normalized recovery curves were fitted to a single exponential relationship: I/Imax = y0 + A1 * (1−exp (-t/τ)), where I is the current amplitude observed at recovery time t, Imax is the maximal current amplitude, y0 is the level of non-inactivating sodium current, A1 is the relative proportion of current recovering from inactivation with a time constant τ.

Experimental data for each investigated cell were fitted by the corresponding function (see above) to yield the relevant parameters, and these were then pooled for statistical comparison. Reported values of membrane potentials were not corrected for a liquid junction potential.

Results are presented as mean ± standard error of the mean (SEM), and statistical comparisons were made between data from mutants and WT sodium channels using Student’s unpaired *t*-test. Statistical significance was set at p < 0.05.

### Prediction of mutation impact and evolution conservation

The effects of the nonsynonymous amino acid change in the seven variants were predicted using two bioinformatics tools: Meta-SNP^59^ and PredictSNP^60^. Both programs combine several tools for predicting variant classification; Meta-SNP has four tools integrated, and PredictSNP uses six different tools. As a reference protein sequence, UniProtKB-P35498 was used in FASTA format. Sequence alignment of the observed patients’ variants and the conservation scoring from 0 for the least conserved, up to 10 for the most conserved position was performed with the programme PRALINE^61^ with the following reference protein sequences used: *SCN1A* (P35498, ENST00000303395.8), *SCN2A* (Q99250, ENST00000283256.10), *SCN3A* (Q9NY46, ENST00000283254.12), *SCN4A* (P35499, ENST00000435607.3), *SCN5A* (Q14524, ENST00000333535.8), *SCN7A* (Q01118, ENST00000643258.1), *SCN8A* (Q9UQD0, ENST00000354534.10), *SCN9A* (Q15858, ENST00000303354.11), *SCN9A* (Q15858, ENST00000303354.11), *SCN10A* (AAD30863, ENST00000449082.3), *SCN11A* (NP 054858, ENST00000302328.8), chimpanzee (A0A2J8PQC8, ENSPTRT00000023334.5), Macaque (A0A1D5QX02, ENSMMUT00000063716.1), mouse (A2APX8, ENSMUST00000112366.7), rat (A0A0G2K6Y2, ENSRNOT00000091259.1), dog (F1PXD7, ENSCAFT00000018258.3), zebrafish (A0A0R4IUM7, ENSDART00000161648.3), electric eel (P02719), pufferfish (BAA90398), drosophila (P35500), and squid (AAA16202).

## Supplementary information

Supplementary information.
