# Supplementary material for: A Study among the Genotype, Functional Alternations, and Phenotype of 9 SCN1A Mutations in Epilepsy Patients
Source: Sci Rep. 2020 Jun 24;10:10288. doi: 10.1038/s41598-020-67215-y (PMC7314844; doi:10.1038/s41598-020-67215-y)
Supplement: Supplementary file 1 — Supplementary information. [file 41598_2020_67215_MOESM1_ESM.pdf]

A Study among the Genotype, Functional Alternations, and Phenotype of 9 *SCN1A* Mutations in Epilepsy Patients

Daniela Kluckova[1], Miriam Kolnikova[2], Lubica Lacinova[3], Bohumila Jurkovicova-Tarabova[3], Tomas Foltan[2], ViktorDemko[4], Ludevit Kadasi[1,5], Andrej Ficek[1], Andrea Soltysova[1,5*]

[1]Department of Molecular Biology, Faculty of Natural Sciences, Comenius University, Ilkovicova 6 Bratislava, 842 15 Slovakia

[2]Department of Pediatric Neurology, Comenius University Medical School and National Institute of Children's Diseases, Bratislava, Slovakia

[3]Center of Biosciences, Institute of Molecular Physiology and Genetics, Slovak Academy of Sciences, Bratislava, Slovakia

[4]Department of Plant Physiology, Faculty of Natural Sciences, Comenius University, Ilkovicova 6 Bratislava, 842 15 Slovakia

[5]Institute for Clinical and Translational Research, Biomedical Research Center, Slovak Academy of Sciences, Dubravska cesta 9, 845 05 Bratislava, Slovakia

***Corresponding author**

Andrea Soltysova, Department of Molecular Biology, Faculty of Natural Sciences, Comenius University, Ilkovicova 6, Bratislava, 842 15 Slovakia, Phone number: +421 2 9014 2050, andrea.soltysova@uniba.sk

ORCID number: Daniela Kluckova 0000-0003-3946-7663
Andrea Soltysova 0000-0002-1402-3583

Supporting information

Supplementary Table 1 Summary of the studied SCN1A variants and used mutagenesis primers.

**Variant**	**Longer transcript ENST00000303395.8**		**Shorter transcript ENST00000637988.1**		**Region**	**Mutagenesis primers (5´ - 3´)**
	cDNA position	Protein change	cDNA position	Protein change		
**D249E**	**c.747T>G**	**p.Asp249Glu**	c.747T>G	p.Asp249Glu	DIS4-S5	GAAGCTCTCAGAGGTAATGATCCT<br>TTCACAGACTGGATCAGGGCT
**E788K**	**c.2362G>A**	**p.Glu788Lys**	c.2329G>A	p.Glu777Lys	DIIS1-S2	ATGGCCATGGAGCACTATCCA<br>GAAAAGAGTATTTAAGACAATACAGATGGT
**W384***	**c.1151G>A**	**p.Trp384***	c.1151G>A	p.Trp384*	DIS5-S6	GACTCAGGACTTCTAGGAAAATCTTTATC<br>ATTAGTCGAAACAAGGACAAAAAAGC
**E78D**	**c.234G>T**	**p.Glu78Asp**	c.234G>T	p.Glu78Asp	N-terminal	CAGAGCCCCTGGATGACCTGGACC<br>ACACCATCTCTGGAGGAATGT
**T1934I**	**c.5801C>T**	**p.Thr1934Ile**	c.5768C>T	p.Thr1923Ile	C-terminal	TTTAAAGCGAATTGTAAAACAAGC<br>AGGTGGCGTCTGTAAGCAC
**R1596C**	**c.4786C>T**	**p.Arg1596Cys**	c.4753C>T	p.Arg1585Cys	DIVS2-S3	AACTCATCTCTCTATGCCATTATTATTT<br>TCAGTACACACTCTCCAGTAAATAGC
**M909K**	**c.2726T>A**	**p.Met909Lys**	c.2693T>A	p.Met898Lys	DIIS5	CCGTGGTCGGCAAGCAGCTCTTTGGT<br>CAAAAATGAAGACGATGATGGCCAAG
**E78***	**c.232G>T**	**p.Glu78***	c.232G>T	p.Glu78*	N-terminal	CAGAGCCCCTGTAGGACCTGGAC<br>ACACCATCTCTGGAGGAATGT
**E1587K**	**c.4759G>A**	**p.Glu1587Lys**	c.4726G>A	p.Glu1576Lys	DIVS2	GCTATTTACTGGAAAGTGTGTACTGAA<br>ACAATGAACACCAGATTGATGCGT
